# Risk prediction and impaired tactile sensory perception among cancer patients during chemotherapy^1^

**DOI:** 10.1590/1518-8345.1979.2957

**Published:** 2018-01-08

**Authors:** Ana Carolina Lima Ramos Cardoso, Diego Dias de Araújo, Tânia Couto Machado Chianca

**Affiliations:** 2 MSc, Technical Manageress, Hope Oncologia, Coronel Fabriciano, MG, Brazil. Doctoral student, Departamento de Enfermagem Básica, Universidade Federal de Minas Gerais, Belo Horizonte, MG, Brazil.; 3 PhD, Departamento de Enfermagem Básica, Universidade Federal de Minas Gerais, Belo Horizonte, MG, Brazil. Professor, Departamento de Enfermagem, Universidade Estadual de Montes Claros, Montes Claros, MG, Brazil.; 4 PhD, Full Professor, Departamento de Enfermagem Básica, Universidade Federal de Minas Gerais, Belo Horizonte, MG, Brazil.

**Keywords:** Neoplasms, Drug Therapy, Neurotoxicity Syndromes, Touch Perception, Nursing Diagnosis

## Abstract

**Objectives:**

to estimate the prevalence of impaired tactile sensory perception, identify risk factors, and establish a risk prediction model among adult patients receiving antineoplastic chemotherapy.

**Method:**

historical cohort study based on information obtained from the medical files of 127 patients cared for in the cancer unit of a private hospital in a city in Minas Gerais, Brazil. Data were analyzed using descriptive and bivariate statistics, with survival and multivariate analysis by Cox regression.

**Results:**

57% of the 127 patients included in the study developed impaired tactile sensory perception. The independent variables that caused significant impact, together with time elapsed from the beginning of treatment up to the onset of the condition, were: bone, hepatic and regional lymph node metastases; alcoholism; palliative chemotherapy; and discomfort in lower limbs.

**Conclusion:**

impaired tactile sensory perception was common among adult patients during chemotherapy, indicating the need to implement interventions designed for early identification and treatment of this condition.

## Introduction

Cancer is a public health problem with a strong impact, as it is the second most prevalent cause of death in Brazil^1^. Its treatment involves interventions to eradicate the tumor, among which antineoplastic chemotherapy (ACH) that acts on cell cycle and cell division, non-selectively interrupting proliferating cells, causing toxicity^2^.

The literature has reported an association between neurotoxicity and the use of various drugs, such as Taxol (Docetaxel and Paclitaxel), Vinca alkaloids (Vincristine, Vinblastine and Vinorelbine), Platinum (Cisplatin, Carboplatin and Oxaliplatin) and Proteasome inhibitors (Bortezomib)^3^. It is suspected that these distinctly compromise important structures of the Peripheral Sensory Nerves (PSN), such as mechanoreceptors, microtubules, axons, and dorsal root ganglion bodies^2^ ^,^ ^4^.

PSN lesions weaken the reception, transmission and response to stimuli, consequently, impairing one’s tactile sensory perception^5^ ^-^ ^7^. These findings are found in clinical practice.

Impaired tactile sensory perception may harm individuals, causing falls, tingling sensations and numbness, pain in the hands and feet, stress and impaired limb function^8^, with the potential to limit the performance of Daily Living Activities (DLA) and interrupt ACH^9^. Note there is increased risk of developing the problem when chemotherapy is associated with abusive alcohol consumption, nutritional problems, Acquired Immunodeficiency Syndrome (AIDS), or other neurotoxic medications.

Studies^6^ ^,^ ^10^ ^-^ ^11^ ^)^ show that the occurrence of altered tactile sensory perception affects from 57% to 98% of cancer patients during treatment with neurotoxic ACH. Nonetheless, the risk factors for this population are not well-established.

Even though impaired tactile sensory perception may affect cancer patients receiving neurotoxic ACH, it can also affect diabetic and alcoholic individuals^12^. The NANDA International (NANDA-I) does not include diagnoses describing this human response.

The diagnose process is essential for nurses to identify appropriate interventions, aiming to achieve positive results such as pain management, safe maintenance, and maximization of patients’ physical function^13^ ^-^ ^14^.

This study is justified by the need to acquire greater knowledge about impaired tactile sensory perception in adult patients receiving ACH, to determine its incidence and risk factors. Such a phenomenon is of interest in the nursing field and its investigation can support the implementation of evidence-based practices to prevent and treat the problem.

This study’s objectives include the estimation of prevalence of impaired tactile sensory perception, identification of risk factors, and the establishment of a risk predictive model among adult patients receiving ACH.

## Method

This is a historical cohort study with analysis of the data contained in the medical files of patients cared for between July 2006 and December 2013 concerning exposure to risk factors and time elapsed from the beginning of treatment up to the onset of impaired tactile sensory perception. The study was conducted in the cancer unit of a medium-sized private hospital located in a city of Minas Gerais, Brazil.

Inclusion criteria were: the medical files of individuals aged 18 years old or older with a malign neoplasia, having a medical indication of ACH, exclusively. The medical files of patients with a documented prior history of impaired tactile sensory perception were excluded.

In the study period, information contained in the files of 253 patients admitted and accessed in the aforementioned oncological unit was analyzed. Of these, 103 did not meet the inclusion criteria, while the files of five patients were not located, so that a final sample of 127 patients was obtained.

The participants had their files analyzed at the time of admission, at every ACH cycle, that is, every month, and at the end of the ACH or when the condition emerged. Two instruments were used to collect data. The first, filled out at the time of admission, addressed sociodemographic and clinical data, prior oncological treatments, and symptomatology of impaired tactile sensory perception. The second instrument was meant to record subsequent assessments that corresponded with the patients’ answers for every ACH cycle, and addressed clinical data and risk factors for the development of this health condition^3^ ^,^ ^5^ ^-^ ^8^ ^,^ ^15^ ^-^ ^16^. Note that information was based on medical and nursing reports contained in the patients’ files. 

The dependent variable was time since admission, that is, when the ACH was initiated up to the onset of the condition, i.e., impaired tactile sensory perception. Independent variables^3^ ^,^ ^5^ ^-^ ^8^ ^,^ ^15^ ^-^ ^16^ were: primary cancer disease; staging; alcoholism; *Diabetes Mellitus*; data concerning ACH, such as protocol, dose, dilution, venous access; medications used; toxicities; skin changes; sensory neurotoxic aspects (sensory neuropathy, paresthesia and dysesthesia) established by Common Toxicity Criteria of Adverse Events (CTCAE)^17^ and sensory aspects addressed in the Antineoplastic Induced Neurotoxicity Questionnaire (AINQ)^9^.

Note that the translated version of the AINQ validated for Brazilian Portuguese^11^ includes the occurrence of neurological changes in both the upper and lower limbs, as well as the person’s ability to perform DLA during ACH. 

Data were doubled entered in the Epi Info, version 3.5.1 to ensure consistency of data, after which, data were exported to the Statistical Package for the Social Sciences (SPSS), version 19.0, for treatment and analysis.

Descriptive analysis was performed with simple frequencies, central tendency measures (mean and median) and variability measure (Standard Deviation - sd). The incidence (global incidence and incidence density) of impaired tactile sensory perception and risk factors were determined.

Bivariate analysis was performed to verify the association of potential risk factors with time since the beginning of treatment up to the emergence of the outcome, using the Chi-square test and Student’s t test. Hence, the relationship between each independent variable and the outcome variable (time since the beginning of the treatment and occurrence of impaired tactile sensory perception) was verified, while the measure of strength of association was verified by Hazard Ratio (HR), considering a Confidence Interval (CI) of 95%.

The Cox proportional hazards model was used to identify the covariates that influenced time since the beginning of treatment up to the onset of the condition. Variables with a p-value ≤0.25 in the bivariate analysis were included in the multivariate analysis model. The global adjustment of the model was obtained by means of the likelihood ratio test^18^.

This study complies with Resolution 466/12, which regulates research with human subjects, while the project was approved by the Institutional Review Board (CAAE - 192666113.7.0000.5149).

## Results

Among the 127 medical files assessed, 73 presented altered sensory perception. The global incidence was 57% in the study period, corresponding to an incidence rate of three cases for each 1,000 patients, ranging from two to four, with a confidence interval of 95%.

Most (62%) were women aged 55 years old, on average (sd±16 years). Of the 127 patients, 53% were Caucasian, 73% were married, 58% had completed high school, and 50% were homemakers or retired.

Of the total patients, 77% originated from oncological clinics, had a diagnosis of gastrointestinal tract cancer (28.3%), hematological (24.4%), gynecological (22.8%), or lung malignant disorders (12.6%), or disorders in other body systems (9.4%), in addition to unknown primary sites (2.5%). At the time of admission, we verified that 51% of the patients presented distant metastases, that is, had stage IV cancer. After this level, ACH is defined as palliative care^2^. The most common sites of metastasis were the liver (13%), regional lymph nodes (11%), lungs (10%), and peritoneum (9%). Most (80%) patients were receiving the ACH for the first time and the surgeries most frequently reported as prior treatments were lymphadenectomy (57%) and tumor resection (47%).

In most cases, the objective of the ACH was palliative (61%), while peripheral vascular access was the most frequently used (62%) to administer ACH. The most common toxic effects included: nausea (57%), hematological toxicity (49%), oral alterations (48%), neuropathies (46%), and fatigue (30%).

The most common sensory aspects reported through the AINQ included: tingling (42%), numbness (40%), burning pain (20%), discomfort (49%), and a heavy feeling in the lower limbs (23%). In regard to the upper limbs, the patients mainly reported dysesthesia (44%) and paresthesia (43%), discomfort (48%), burning pain (44%), and a heightened sensitivity to touch (14%).

The patients also reported decreased sensitivity (54%), lack of/decreased touch sensitivity (44%), feeling of stepping on sand (21%), stinging in the lower limbs (19%), and numbness (18%). Some patients reported they needed assistance to walk (13%), while others reported problems performing manual tasks (12%).

The bivariate analysis presented variables that were statistically (p≤0.25) associated with time since the beginning of treatment up to the onset of impaired tactile sensory perception. A total of 80 variables were eligible for the multivariate analysis, 33 of which were statistically significant (p<0.05) and are presented in Table 1.

**Table 1:** Variables associated with time since the beginning of treatment up to the onset of impaired tactile sensory perception. Coronel Fabriciano, MG, Brazil, 2006-2013

Variables	Impaired tactile sensory perception						HR*[CI 95%^†^]	p^‡^
	Yes		No		Total			
	n	%	n	%	n	%		
Hepatic metastasis	4	5	1	2	5	4	5.26 [1.85-14.9]	0.002
Palliative chemotherapy	35	48	42	78	77	61	0.52 [0.32-0.83]	0.007
Phlebitis	6	11	14	19	20	16	2.05 [1.12-3.75]	0.021
Peripheral venous access	36	50	41	79	77	62	0.56 [0.35-0.90]	0.017
6^th^ cycle of chemotherapy	14	19	24	44	38	30	0.23 [0.07-0.70]	0.01
Oxaliplatin	26	36	7	13	33	26	1.63 [1.00-2.65]	0.049
Carboplatin	8	11	14	26	22	17	0.40 [0.18-0.89]	0.025
Saline solution to prepare chemotherapy	18	25	2	4	20	16	1.85 [1.08-3.17]	0.026
Peripheral sensory neuropathy degree 1	27	37	1	2	28	22	6.45 [3.40-12.24]	<0.001
Peripheral sensory neuropathy degree 2	21	29	0	0	21	17	4.13 [2.12-8.03]	<0.001
Peripheral sensory neuropathy degree 3	8	11	0	0	8	0.6	3.38 [1.43-8.02]	0.006
Clinical manifestation in lower limbs (AINQ^||^)								
Discomfort without contact with ice	51	70	6	11	57	45	3.18 [1.83-5.51]	<0.001
Tingling (paresthesia) degree 1	26	36	0	0	26	20	3.79 [2.07-6.97]	<0.001
Tingling (paresthesia) degree 2	14	19	0	0	14	11	3.06 [1.52-6.19]	0.002
Tingling (paresthesia) degree 3	6	8	0	0	6	5	3.26 [1.28-8.29]	0.013
Tingling after contact with ice	8	11	0	0	8	6	7.80 [3.35-18.14]	<0.001
Clinical manifestation in upper limbs (AINQ^||^)								
Tingling (paresthesia) degree 1	22	30	1	2	23	18	2.75 [1.48-5.11]	0.001
Tingling (paresthesia) degree 2	24	33	0	0	24	19	3.67 [2.04-6.61]	<0.001
Tingling (paresthesia) degree 3	5	7	0	0	5	4	3.02 [1.12-8.16]	0.029
Numbness (dysesthesia) degree 1	24	33	0	0	24	19	2.76 [1.57-5.01]	0.001
Numbness (dysesthesia) degree 2	22	30	0	0	22	17	3.46 [1.91-6.26]	<0.001
Difficulties feeling cold	10	14	0	0	10	8	2.18 [1.11 -4.31]	0.024
Heightened sensitivity to touch	6	8	0	0	6	5	2.61 [1.12-6.06]	0.026
Discomfort after contact with ice	5	7	0	0	5	4	3.24 [1.16-9.05]	0.025
Other clinical manifestations								
Difficulties when touching cold things	16	22	1	2	17	13	1.79 [1.02-3.12]	0.041
Heightened sensitivity to touch	18	25	0	0	18	14	2.65 [1.54-4.57]	<0.001
Peripheral discomfort after contact with ice	13	18	1	2	14	11	5.06 [2.39-10.71]	<0.001
Peripheral discomfort without contact with ice	45	62	2	4	47	37	4.15 [2.31-7.47]	<0.001
Lack of/decreased touch sensitivity	56	77	0	0	56	44	3.64 [2.11-6.30]	<0.001
Decreased sensitivity	68	93	0	0	68	54	13.27 [5.35-32.96]	<0.001
Difficulties performing manual tasks	14	19	1	2	15	12	2.08 [1.16-3.75]	0.015
Feeling as if stepping on sand	27	37	0	0	27	21	1.96 [1.21-3.16]	0.006
Stinging/stabbing pain in upper limbs	23	32	1	2	24	19	3.16 [1.91-5.23]	<0.001

*Hazard Ratio; †Confidence Interval; ‡Chi-square;; ||Antineoplastic Induced Neurotoxicity Questionnaire.

A predictive risk model for tactile sensory perception among adult patients receiving ACH resulted from the multivariate analysis (Table 2). Note that among the factors identified, bone, hepatic and lymph regional node metastasis, in addition to palliative chemotherapy, and discomfort in the lower limbs, presented significant impact, along with time elapsed from the beginning of the treatment up to the onset of impaired tactile sensory perception.

**Table 2:** Risk prediction model with time elapsed from the beginning of treatment up to the onset of impaired tactile sensory perception. Coronel Fabriciano, MG, Brazil, 2015

Variables	HR*	CI 95%^†^	p^‡^
Metastases			
Bone	0.04	0.01-0.39	0.004
Hepatic	10.4	3.75-28.7	<0.001
Regional lymph node	0.26	0.07-0.95	0.041
Alcoholism	10.9	2.66-44.5	0.001
Palliative chemotherapy	0.52	0.27-0.93	0.029
Discomfort in lower limbs	3.56	1.69-7.47	0.001

*Hazard Ratio; ^†^Confidence Interval; ^‡^Chi-square

## Discussion

Impaired tactile sensory perception is a subjective problem, that is, patients need to communicate their symptoms, which should be highlighted as a recommendation when care is provided to cancer patients receiving neurotoxic ACH^11^. A total of 57% of the population under study presented altered tactile sensory perception and reported physical weariness due to the treatment including paresthesia in both hands and feet, discomfort in the upper limbs, lack of or decreased touch sensitivity and hypoesthesia, situations widely reported in the literature^5^ ^-^ ^9^ ^,^ ^11^. The rates of impaired tactile sensory perception reported in the literature range from 51% and 98%^6^ ^-^ ^7^ ^,^ ^10^ ^-^ ^11^, depending on the type of medication involved.

The variables presented in this study as factors that predispose cancer patients receiving ACH to a lower risk for the development of the outcome under study were: palliative ACH; peripheral venous access to administer ACH; sixth chemotherapy cycle; and the use of carboplatin. These findings diverge from those reported in the literature, which suggest a relationship between the onset of impaired tactile sensory perception in patients receiving palliative ACH such as paresthesia and dysesthesia, and the cumulative effect of the medications used^6^ ^,^ ^9^ ^,^ ^11^. Among the variables that presented significant association (p<0.05) with time elapsed from the beginning of the treatment and the onset of impaired tactile sensory perception, and which predispose patients to a greater risk of developing the outcome under study (HR≥1), researchers^5^ ^-^ ^11^ ^,^ ^19^ report the use of Oxaliplatin, the emergence of Peripheral Sensory Neuropathy (PSN), report discomfort in lower and upper limbs when in contact with ice, paresthesia, dysesthesia, peripheral discomfort, lack of/decreased touch and sensitivity, and difficulty in performing manual tasks.

Note that the use of Oxaliplatin (HR=1.63, p=0.049) in this study is similar to third generation platinum, commonly used with patients with colon cancer. It promotes neurotoxicity, leading to important loss or altered sensory situations such as decreased touch perception^9^. In this sense, nurses should be attentive to include an assessment of the integrity of patients’ sensory function in the nursing care plan of those receiving Oxaliplatin^20^.

ACH induced sensory neuropathy, especially degree one neuropathy, was identified as one of the main risk factors for impaired tactile sensory perception (HR=6.45, p<0.001). One recent study^10^, however, reports that deficient tactile sensation was a predictor of chemotherapy-induced peripheral neuropathy in patients who received Oxaliplatin to treat colorectal cancer.

In this study, complaints concerning lack of or decreased touch sensitivity manifested by the patients emerged as important risk factors to be identified whenever impaired tactile sensory perception is identified, as they reflect damage caused to structures essential for receiving and transporting electrical impulses, which can be caused by ACH. The interruption of electrical impulses is the main characteristic of ACH-induced sensory neuropathy, toxicity basically caused by axonopathy^5^ ^-^ ^6^ ^,^ ^9^. These results indicate that patients receiving neurotoxic ACH often experience ACH-induced sensory neuropathy and that such a tactile disturbance is the individual’s response to such toxicity. 

In regard to carboplatin, the results are similar to those presented in the literature. This platinum is known for having lower neurotoxicity potential^21^. Neurological symptoms only occur when ACH is administered in high doses, in elderly patients, associated with taxon, or in individuals with prior exposure to another drug with neurotoxic effects^3^.

The multivariate analysis revealed six co-variables that were statistically significant (p<0.05) for time since the beginning of treatment up to the onset of impaired tactile sensory perception. The following were the variables presenting the lowest risk (HR<1) were bone metastases and regional lymph nodes metastases, in addition to palliative ACH; those with the highest risk (HR>1) were alcoholism, discomfort in lower limbs, and hepatic metastasis.

According to the literature^6^ ^,^ ^9^ ^,^ ^11^, the objective of palliative ACH is to improve the quality of life of metastatic patients; however, these patients often receive multiple medications, increasing the risk of cumulative toxic effects, and, therefore, may present more intense responses. Despite the statistical significance found here, further studies are needed to verify whether there is a direct causal relationship between the emergence of metastases and the development of the outcome under study, as data indicate that patients with a more severe clinical profile presented lower risk of impaired tactile sensory perception.

Additionally, authors note that metastases possibly have a compressor effect on peripheral nervous terminations, which may result in tactile alterations, such as paresthesia, dysesthesia and even complete loss of touch sensitivity ^22^.

In regard to alcoholism, the abusive consumption of alcohol, associated with neurotoxic substances, has a known deleterious effect on the peripheral nervous system, worsening the condition of a patient during treatment^5^. In this sense, increased surveillance on the part of the nursing staff is needed with patients who abuse alcohol while under treatment with neurotoxic ACH.

Discomfort in the lower limbs (HR=3.56) appeared as one of the factors with the highest risk of impaired tactile sensory perception. The use of neurotoxic substances during ACH, may damage long fibers and cause symptoms, initially in the lower limbs, also interfering in one’s ability to walk, as reported by several studies^5^ ^-^ ^7^ ^,^ ^10^ ^-^ ^11^ conducted with patients being treated with these substances.

The risk factors for the outcomes identified in this study are related to the clinical conditions of patients, cancer aspects, and treatment with ACH. Even though the nursing staff cannot change such factors, strategies can be adopted that facilitate early identification of impaired tactile sensory perception among patients, as well as implementing preventive measures in order to avoid worse consequences.

The model obtained here was considered valid to describe the relationship existing between time since the beginning of treatment up to the onset of impaired tactile sensory perception and associated risk factors, in addition to predicting which oncological patients are at risk of experiencing impaired tactile sensory perception during ACH, aiding the implementation of nursing care to prevent or treat it.

Despite evidence that cancer patients may present impaired tactile sensory perception during ACH, one has to consider limitations inherent to the use of data contained in medical files and to understand there is a need for multicenter studies to be conducted in different populations, in order to establish external validity.

## Conclusion

The results presented here reveal that impaired tactile sensory perception is a common event among adult cancer patients receiving potentially neurotoxic ACH, especially when Oxaliplatin is used.

After the bivariate analysis and the adjustment stage of the multivariate analysis, the factors that remained as the best predictors for the phenomenon under study, among demographic and clinical factors, were: bone; hepatic and regional lymph node metastases; alcoholism; palliative chemotherapy; and discomfort in lower limbs.

The evidence is that ACH may cause adverse and toxic effects. Therefore, this study contributes to the clarification of impaired tactile sensory perception in patients with cancer receiving ACH, in addition to establishing risk factors. The early identification of risk factors and early implementation of interventions to identify, prevent or treat the problem, can directly contribute to decreasing discomfort and sensory damage that limits the performance of daily activities and compromises quality of life.

This study’s findings, together with those reported by other studies, is a step toward the NANDA-I’s Committee of Development of Diagnoses considering impaired tactile sensory perception as a nursing diagnosis for possible inclusion in the taxonomy.
